# Unleashing Intrapreneurial Behavior: Exploring Configurations of Influencing Factors among Grassroots Employees

**DOI:** 10.3390/bs13090724

**Published:** 2023-08-30

**Authors:** Di Ye, Wenlong Xie, Linlin Zheng

**Affiliations:** 1 College of Business Administration, Huaqiao University, Quanzhou 362000, China; 2 Oriental Enterprise Management Research Center, Huaqiao University, Quanzhou 362000, China

**Keywords:** employees’ intrapreneurial behavior, fuzzy set qualitative comparative analysis, employees’ individual factors, organizational factors

## Abstract

Effectively promoting employees’ intrapreneurial behavior has become the focus of enterprises. This study takes the middle and grassroots employees in enterprises as subjects and explores the configuration effect of multiple influencing factors on employees’ intrapreneurial behavior. Based on employee expectation theory and individual-environment matching theory, this study collates six influencing factors: entrepreneurial self-efficacy, entrepreneurial competence, task school level, perceived value, management support, and reward mechanism. A total of 163 samples were obtained, and the qualitative comparative analysis method based on fuzzy set was used to analyze the influence mechanism and result path of employees’ intrapreneurial behavior from the perspective of the interaction between individual factors and organizational factors. Six influencing paths of employees’ high intrapreneurial behavior were found, which can be divided into ability-driven and value-driven factors, revealing that the six factors can produce equivalent results in different configurations. Furthermore, five influencing paths of employees’ non-high intrapreneurial behavior were divided into three types: ability obstacle type, perception obstacle type, and value obstacle type. These have an asymmetric causal relationship with employees’ high intrapreneurial behavior. This study provides management support for effectively stimulating employees’ intrapreneurial behavior.

## 1. Introduction

In the context of emphasizing the input and output of innovation, innovation capability naturally becomes a crucial guarantee for businesses to maintain competitiveness and achieve positive performance growth. Simultaneously, employees, as carriers of innovation activities within a company, play a key role in utilizing the company’s business resources for innovative activities. With the increasing flattening of organizational structures and the knowledge-based nature of labor in society, the management philosophy used to emphasize hierarchy and authority has gradually shifted toward emphasizing equality and cooperation. The focus has shifted from a supervision-oriented “economic person” to an achievement-oriented “entrepreneur”. They are willing to take risks, are enthusiastic about facing challenges and overcoming difficulties, and passionate about value creation. Many companies encourage employees with entrepreneurial intentions to undertake new businesses and projects within the organization. In this approach, employees are primarily responsible for the entire business process, and share profits and achievements with the company during the business settlement. This approach is known as the internal entrepreneurship mode. It not only satisfies employees’ entrepreneurial desires, but also stimulates the vitality of internal personnel and enhances the company’s innovation capabilities.

Google encourages its employees to document their innovative ideas. The company provides funding and technical support, allowing employees to dedicate 20% of their working time to implementing ideas [1]. This approach not only meets the individual needs of employees, but also enhances the company’s internal innovation capacity. Huawei offers supportive policies and preferential treatment for entrepreneurial employees. They provide equipment and promise to reassign employees to other positions within the company if their entrepreneurial endeavors fail within six months. This not only addresses employees’ concerns about their future but also enables them to focus on intrapreneurial activities [2]. As a result, it facilitates the creation of new businesses and ventures for the company. Research has confirmed that internal entrepreneurship has a positive impact on employees’ work engagement through the mediating variable of psychological capital. Management can encourage employees’ intrapreneurial behavior, which can lead to wholehearted dedication to work [3]. Moreover, high consistency between user-driven innovation and employee intrapreneurship as sources of higher innovation performance has been confirmed [4]. Thus, at the organizational level, employees’ entrepreneurial behavior has gradually become an important way for companies to enhance their innovation capabilities and maintain core competitiveness.

Intrapreneurial behavior is a process in which employees, based on new employment opportunities, utilize the platform provided by their parent company to create new ventures and drive overall strategic updates within the company [5]. However, employees’ intrapreneurial behavior does not always occur as expected by managers, as not all employees respond to a company’s call to engage in internal entrepreneurship. Employees who lack the corresponding traits or environmental stimuli may not exhibit entrepreneurial behavior [6]. It is important to study how to motivate employees to engage in intrapreneurial behavior and identify to employee teams suitable for internal entrepreneurship; these factors play a significant role in the innovative development of a company. Currently, the academic community has conducted extensive research on the concept of internal entrepreneurship, including internal entrepreneurship within companies and among middle- and senior-level managers. However, relatively less research specifically focuses on employee-level intrapreneurial behavior. Previous research has found that individual factors such as personality traits and abilities, as well as organizational factors such as leadership style and team type, impact employees’ intrapreneurial behavior. However, most studies have focused on exploring the impact of single factors or the interactive effects between two or three variables on intrapreneurial behavior. In reality, employees’ intrapreneurial behavior is influenced by a complex interplay of multiple factors. Different combinations of factors can lead to varying outcomes. Therefore, studying intrapreneurial behavior from a single perspective may result in research biases, highlighting the need for a holistic approach.

This study focuses on the core concept of the conditions for employees’ intrapreneurial behavior. We conducted a comprehensive review of the existing literature and integrated the theory of person-environment fit to construct a research model that considers both individual and organizational factors influencing employee behavior. Furthermore, we adopted an employee expectancy theory perspective and analyzed six factors influencing employees’ intrapreneurial behavior based on the basic formula of motivational force in expectancy theory and existing research in the field of internal entrepreneurship. Through a qualitative comparative analysis (QCA) based on fuzzy sets, we examined the driving mechanisms of employees’ intrapreneurial behavior, explored effective paths and the necessary and core conditions for such behavior, and further analyzed the reasons for different factors playing different roles in various paths. This expands the research on employee-level intrapreneurial behavior and provides management insights into driving employees’ intrapreneurial behavior and enhancing innovation capabilities in organizations.

This study employs the fuzzy-set-based QCA method (fsQCA) to analyze the configurations of the antecedent variables involved in the research framework. This approach provides a systematic explanation of the causal relationships in complex problems and reveals the influence of multiple-path results on employees’ internal entrepreneurship from a holistic perspective. Compared to previous studies that focused on single-level or linearly examined individual factors, the exploration of the configuration effects of multiple antecedent variables allows for a clearer and more profound understanding of the mechanisms driving employees’ internal entrepreneurship.

## 2. Literature Review and Theoretical Background

### 2.1. Employees’ Intrapreneurship

The concept of intrapreneurship originally referred to a process in which relatively independent small teams within large organizations or companies engaged in entrepreneurial activities. Fischer defines intrapreneurship as the actions taken by established companies to enhance profitability, achieve strategic renewal, and drive innovation [6]. Moreover, intrapreneurship is defined as activities undertaken by an organization to strengthen innovation, assume risks, and proactively respond to environmental forces [7]. Others define it from the perspective of individual employees, stating that it involves employees within an organization deviating from routine to pursue new opportunities, engage in new activities, or take bottom-up, work-related proactive actions [8]. Additionally, it is described as the proactive work-related activities of capable employees who can transform ideas into business success using a bottom-up approach [9]. Currently, a widely accepted comprehensive concept of intrapreneurship emphasizes the differentiation between its organizational and individual aspects [5]. By integrating both personal and organizational aspects, the following definition is proposed: Intrapreneurship refers to the process in which employees identify and exploit opportunities through innovation, proactiveness, and risk-taking, enabling the organization to create new products, processes, and services, and initiate self-renewal or entrepreneurship to enhance the organization’s competitiveness and performance [5]. Other scholars argue that intrapreneurship, regardless of the size of the company, has a significant impact on various aspects of organizational development. They believe that intrapreneurship is essentially an activity-driven concept that leads the organization toward new directions in terms of its products and services, technology, structure, or operations [10,11,12]. Employees are identified as initiators and primary contributors to the innovation process, and innovative employee behavior is driven by intrapreneurship (bottom-up) [13,14].

The current consensus in academia is that intrapreneurship exists at two levels: organizational and individual. Organizational-level intrapreneurship is considered to be more aligned with entrepreneurial activities at the company level, with initiators being predominantly top-level managers who emphasize the creation of new ventures. Therefore, organizational-level intrapreneurship bears a strong resemblance to corporate entrepreneurship. By contrast, individual-level employee intrapreneurship primarily focuses on non-managerial or lower-level managerial employees [15]. Employees often passively perform job tasks and instructions. In individual-level intrapreneurship, employees take responsibility for innovation, and proactively and spontaneously pursue internal entrepreneurial projects. This is referred to as bottom-up intrapreneurship.

### 2.2. Factors Influencing Employees’ Intrapreneurial Behavior

Employee intrapreneurship focuses on the individual rather than the organizational level. It emphasizes the proactive assumption of innovation responsibilities by non-managerial or lower-level managerial employees within the existing organizational framework, engaging in bottom-up entrepreneurial activities. Employees are constantly situated within the organization, and, according to the person-environment fit theory, their behavior is influenced not only by individual factors but also by their organizational environment. A combination of these factors determines the subsequent employee behavior. Therefore, the factors that influence employee intrapreneurship include both individual and organizational factors. Individual factors primarily serve as intrinsic motivations for employee intrapreneurship, while organizational factors act as external influences.

#### 2.2.1. Employees’ Individual Factors

Individual traits, abilities, and cognition are important factors that influence employee intrapreneurship. Individual traits and human capital influence intrapreneurial employee behavior [16]. A comprehensive capability indicator known as entrepreneurial competence is a key factor in determining employee intrapreneurship behavior [17]. Scholars have elucidated the content of entrepreneurial competence, which includes opportunity perception, risk taking, and resource allocation abilities. Based on these criteria, entrepreneurial competence can be divided into self-perception and practical ability. Employees with high self-efficacy perceptions often exhibit intrapreneurial behaviors. By contrast, in terms of practical ability, opportunity recognition ability significantly enhances the likelihood of employees engaging in intrapreneurial behavior [18]. Regarding cognitive factors at the employee level, employees’ organizational identification, which refers to their sense of belonging to an organization, is positively correlated with the occurrence of intrapreneurial employee behavior [19]. Additionally, employees’ organizational identification partially mediates the relationship between leadership and intrapreneurial behavior [19]. Employee job satisfaction plays a significant role in the process of intrapreneurial behavior. High job satisfaction has a positive impact on intrapreneurial behavior within an organization [20,21]. Scholars have introduced the concept of self-efficacy at the employee cognitive level [22,23]. Self-efficacy refers to employees’ cognitive beliefs about their ability to accomplish a specific task successfully. They found a relationship between self-efficacy and employees’ intrapreneurial spirit, indicating that higher self-efficacy is associated with a higher likelihood of intrapreneurial behavior [22,23]. Obtaining management support is crucial for employees willing to engage in intrapreneurial activities. Management support includes managerial behaviors that foster employees’ intrapreneurial spirit, such as encouraging employees and providing them with appropriate assistance [24].

#### 2.2.2. Organizational Factors

Organizational flexibility can provide employees with a smoother and more diverse information flow [25]. High flexibility increases the likelihood of entrepreneurial behavior among employees. Organizational flexibility is positively correlated with employees’ job satisfaction and self-efficacy [25]. Allowing employees to design their own work leads to more entrepreneurial activities, thereby positively influencing their intrapreneurial behavior [26]. Simultaneously, employees’ job autonomy enhances their self-efficacy [25]. Organizational rewards and reinforcements constitute the fourth dimension. Rewards can effectively increase employees’ willingness to participate in innovative projects [27].

Scholars have researched internal entrepreneurship from various perspectives including individual abilities, employee cognition, organizational structure, management styles, and social influence. Currently, most scholars focus on one aspect to draw corresponding conclusions, overlooking the fact that the emergence of employee behavior is not solely determined by individual factors, but is closely related to the organizational environment. Multiple factors influence the decisive factors in subsequent employee behavior. Therefore, conclusions derived from linear studies of one-dimensional factors lack comprehensive consideration, and further in-depth research is required in this area.

### 2.3. The Theory of Person-Environment Fit

Person-environment fit theory suggests that the degree of alignment between individuals and their environments greatly influences employees’ job attitudes and subsequent behaviors. This theory explores the outcomes and impacts that arise during a match or mismatch between an individual’s values, expectations, goals, and the environment. Scholars consider the results of person-environment fit to be predictive indicators of employees’ job attitudes and subsequent behaviors [28,29]. In the context of intrapreneurial behavior, employees’ judgment of engaging in such behavior is not solely influenced by individual factors, but also by changes in their surrounding environment. When employees perceive that they are unable to achieve their goals or that environmental obstacles are too significant for them to benefit from internal entrepreneurship, they are likely to refrain from engaging in such behaviors. Conversely, during favorable conditions, employees are more likely to proactively seek internal entrepreneurial opportunities and engage in such activities. Therefore, when exploring the factors that influence employees’ intrapreneurial behavior, it is crucial to consider both the individual characteristics of employees and their organizational environment. Only by considering both aspects can we gain a more comprehensive understanding of intrapreneurial behavior, and the theory of person-environment fit can effectively support the factors influencing employees’ intrapreneurial behavior.

### 2.4. The Theory of Employee Expectations

Regarding employee expectations, expectancy theory was initially proposed by American psychologist Vroom [30]. The author suggests that individuals have different needs, and the existence of these needs continuously stimulates their intrinsic motivation to fulfill various needs by achieving their goals. When individuals assess if something meets their internal expectations, they consider the probability of accomplishing the event, and the value derived from its completion. The basic expectation model is expressed as motivation (M) = expectancy (E) × valence (V). According to expectancy theory, when an individual’s expectations and valence regarding a task are high, they experience greater motivation, actively striving to engage in the expected behavior to fulfill their expectations. Conversely, when an individual’s expectations or valence regarding a task are low, the motivational effect diminishes, and they may approach the task with relative passivity, or even experience negative emotions [31,32]. As a form of employee behavior, internal entrepreneurship is the result of a comprehensive evaluation of individual conditions and the organizational environment. This behavior can be explained and predicted by an individual’s expectations and valence. When employees are in favorable situations, they experience strong motivating forces that lead to innovative behavior. Conversely, internal entrepreneurship is inhibited in unfavorable environments. Therefore, the expectancy theory effectively helps us understand the “pathway” of employees’ internal entrepreneurship behavior and to explore the antecedents that influence its occurrence.

## 3. Research Model

**Figure 1:**
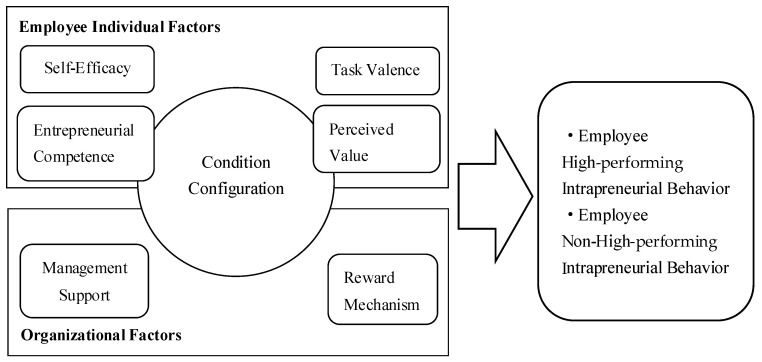
Research Model: Model of Drivers Influencing Employees’ Intrapreneurial Behavior.

Person-environment fit theory suggests that employees exhibit more proactive behaviors when working in environments that match their personal characteristics. The interaction between individual characteristics and environmental factors influences employees’ subsequent behaviors [30,33]. Therefore, internal entrepreneurship among employees is influenced by the organizational environment. Building upon person-environment fit theory, this study explores the antecedents that influence employees’ internal entrepreneurship by examining both individual and organizational management factors [34]. As a form of individual activity conducted within an organization, intrapreneurial behavior is influenced by individual conditions and organizational environmental factors. This can be attributed to the magnitude of the driving forces exerted by individuals. According to expectancy theory, individuals are driven by a desire to satisfy certain needs and strive to achieve their goals. When a goal is unfulfilled, individuals develop expectations that generate a motivating force, thereby mobilizing their enthusiasm and promoting subsequent behaviors. The motivating force experienced by individuals can be expressed as the product of expectations (E) and valence (V): motivation (M) = expectancy (E) × valence (V) = expectancy (E1 × E2) × valence (V). E represents expectancy, which is the perceived likelihood of achieving personal goals, and V represents valence, which is an individual’s perception of the value of rewards. E1 represents the expectancy of organizational goals, indicating the probability of individuals achieving organizational goals. E2 represents the expectancy of personal goals, indicating the probability of receiving rewards through job performance. Based on the fundamental formula, the greater the motivating force experienced by an individual, the higher their enthusiasm for the corresponding behaviors. The motivating force is directly related to expectancy values (E1 and E2) and valence (V), and the product of these three factors determines the magnitude of employee motivation. When all three factors are at high levels, motivation is stronger and employees are more likely to actively complete organizational tasks. Conversely, when any of the factors are at a low level, the generated motivation decreases and employees tend to respond more passively to organizational tasks. The three-level relationship corresponding to expectancy theory, namely individual effort and performance, performance and organizational rewards, and organizational rewards and individual goals, directly determine the magnitude of the motivating force received by employees, thereby influencing the occurrence of employees’ intrapreneurial behavior [35]. Therefore, it is highly feasible to explore and extract the factors influencing employees’ internal entrepreneurship by studying the specific logic behind these three-level relationships. Based on the three-level relationship implied by expectancy theory and the existing research overview of employees’ internal entrepreneurship, this study identifies specific influencing factors at the individual and organizational levels in the research model. Based on the person-environment fit theory and the motivation equation of expectancy theory, the factors influencing employees’ intrapreneurial behavior are analyzed from three levels: organizational goal expectations, individual goal expectations, and valence. The core factors of this research model are identified as entrepreneurial self-efficacy, entrepreneurial competence, task valence, perceived value, management support, and reward mechanisms. The research model is shown in Figure 1.

## 4. Methodology

QCA is a set-theoretical analytical method based on Boolean algebra. It explores how complex social problems occur by analyzing the sufficient and necessary relationships between antecedent variables and outcomes as a whole. In organizational and management research, QCA provides a new approach to explain the complex causal relationships of organizational practices, including concurrent causality, asymmetry, and equifinality [36,37]. To investigate the impact mechanism of antecedent factors such as entrepreneurial competence and managerial support on employee intrapreneurial behavior, this study adopts a fsQCA method selected based on the following considerations:

First, QCA conceptualizes the relationship between antecedent and outcome variables as complex causal relationships, rather than simple linear relationships. Employees’ intrapreneurial behavior, an an important means of enhancing individual motivation and organizational innovation capabilities, is inherently complex. Its occurrence is not influenced by a single factor, but by the combined effects of multiple antecedent factors [36]. Therefore, traditional quantitative analysis based on binary relationships between independent and dependent variables is insufficient to explain the underlying mechanisms of intrapreneurial employee behavior. FsQCA, however, emphasizes the complexity of causal relationships and explores the interactions between antecedent factors to uncover the paths that lead to the outcomes.

Second, QCA focuses on exploring the multiple concurrent paths that lead to the occurrence of outcomes, known as equifinality, in causal configurations [37,38,39]. Since there is no unique combination of antecedent conditions that leads to a specific outcome, multiple combinations of antecedent conditions can lead to the same outcome. The configurational perspective of QCA acknowledges the existence of multiple configurations that can produce the same outcome and allows for a holistic analysis of combinations of antecedent conditions that result in a particular outcome. A review of the relevant literature on employee intrapreneurship reveals a lack of uniqueness in the combination of antecedent factors that influence employee intrapreneurship. FsQCA recognizes the equifinality of different causal configurations, which is highly relevant to this study.

Third, QCA emphasizes the asymmetry of the causal relationships. Traditional empirical analysis methods often assume that antecedent variables are necessary and sufficient conditions for outcome variables. However, a review of the relevant literature reveals that in the process of employee intrapreneurship, individual and organizational driving factors may have different effects on the occurrence and degree of behavior, leading to variations in the combinations of antecedent variables that result in different levels of the outcome variable. Asymmetrical causal relationships can be handled better by QCA, facilitating an understanding of the relationships between antecedent and outcome variables.

### 4.1. Variable Definition and Measurement

Based on a review and synthesis of the relevant research in the field of intrapreneurship, this study selects six variables as antecedent factors influencing employees’ intrapreneurial behavior. These variables are categorized into two levels: individual and organizational. At the individual level, the selected variables were entrepreneurial competence, self-efficacy, task valence, and perceived value. At the organizational level, the selected variables are managerial support and reward mechanisms. The outcome variable in this study is employees’ intrapreneurial behavior. By examining the relationships between these variables, this study aims to uncover the driving mechanisms that influence employees’ intrapreneurial behavior.

#### 4.1.1. Measurement of Independent Variables

1. Entrepreneurial competence: Entrepreneurial competence is considered an important factor in determining employees’ intrapreneurial behavior. It is divided into two parts: opportunity identification ability and operational management ability, comprising 16 items [40].
2. Entrepreneurial self-efficacy: Entrepreneurial Self-efficacy refers to employees’ perceptions of their ability to successfully accomplish a task. It has three dimensions: risk tolerance, relationship management, and others. It comprises of nine measurement items [41].
3. Task Valence: Task valence represents employees’ subjective judgments of the rewards they can obtain after completing their intrapreneurial activities. The concept of task efficacy is applied less frequently at the employee level [42].
4. Perceived Value: Perceived value refers to employees’ cognitive perceptions of the value of rewards and compensation [43,44].
5. Managerial Support: Managerial support refers to the comprehensive support provided by an organization or company for innovative employee behavior. It is an important factor at the organizational level that influences employees’ intrapreneurial behavior and has a significant promoting effect on their willingness and enthusiasm for intrapreneurship [45].
6. Reward mechanism: Reward mechanism represents an organization’s use of the corresponding reward reinforcement to provide employees with material or psychological rewards [46].

#### 4.1.2. Measurement of Dependent Variables

Employee intrapreneurial behavior: Employee intrapreneurial behavior is a multilevel and comprehensive concept that is primarily based on a scale [47] to measure individual employees’ intrapreneurial behavior.

### 4.2. Materials and Methods

Based on the definition of intrapreneurial behavior, it is evident that employees identify and exploit opportunities through innovation, proactiveness, and risk-taking to enable organizations to create new products, processes, and services, and enhance their competitiveness and performance through self-renewal or entrepreneurship [5]. This process is predominantly driven by employees or middle-level managers, representing a bottom-up approach to innovation, rather than by top-level managers. Therefore, the survey primarily targets regular employees and middle-level managers in the organization to ensure sample representativeness, and to facilitate data collection and analysis. To ensure universality of the samples and facilitate the collection and organization of data, surveys will be conducted through the Credamo specialized data platform. After the questionnaire was designed, distributed, and collected, 218 questionnaires were returned, out of which 163 were deemed valid, resulting in a valid response rate of 74.8%.

#### 4.2.1. Descriptive Statistical Analysis of Data

The collected sample data were subjected to statistical analysis focusing on several aspects of the participants’ basic information, including sex, age, education level, and job position. There were 85 male respondents, accounting for 52.1% of the total sample, and 78 female respondents, accounting for 47.9% of the total sample. The sex distribution was relatively balanced, with no significant difference; participants in the age groups 20–30 and 31–40 constituted the majority, with 73 and 74 individuals, respectively. Thus, participants were predominantly young. Most participants (84.1%) held a bachelor’s degree or higher. Approximately 42.9% of participants were classified as middle-level managers, representing nearly half of the sample. The number of regular employees and middle-level managers are approximately equal, which aligns with the research requirements for a participant hierarchy.

#### 4.2.2. Data Validity and Reliability Testing

Cronbach’s alpha coefficient was used to examine the reliability of the questionnaire. The SPSS software was used to calculate this coefficient, and the results showed that the overall Cronbach’s alpha coefficient for the entire scale was 0.924, which was higher than the standard value of 0.7 (see Table 1). Additionally, Cronbach’s alpha coefficients for all subscales ranged from 0.754 to 0.884, exceeding the standard value. These data indicated that the questionnaire had good reliability. The composite reliability (CR) values for this model are all significantly greater than 0.6 (see Table 2), indicating a high level of internal consistency among the items within each construct. This suggests that the items that are indicators of a specific construct are consistent in their representation of that construct. Additionally, the average variance extracted (AVE) values for this model exceed 0.5, demonstrating a satisfactory level of convergent validity.

The Kaiser–Meyer–Olkin (KMO) value for the questionnaire on factors influencing employees’ intrapreneurial behavior was 0.893 (see Table 3), and Bartlett’s test of sphericity was significant, indicating its suitability for factor analysis. Similarly, the KMO value for the questionnaire on employees’ intrapreneurial behavior was 0.849, and Bartlett’s test of sphericity showed a significant level, indicating its suitability for factor analysis as well. Based on the premise that both questionnaires are suitable for factor analysis, this study conducted a validity test on the questionnaires for factors influencing employees’ intrapreneurial behavior and that for employees’ intrapreneurial behavior. The data were imported into the AMOS software for analysis. Standardized loadings of the items for each variable are within the range of 0.6–0.9, with no cross-loadings. Overall, this indicates that the questionnaires have good validity.

#### 4.2.3. Necessity Analysis and Constructing a Truth Table

This study conducted a necessity analysis of the six preconditions of employees’ intrapreneurial behavior using fsQCA3.0. The results indicate no consistency coefficients for the preconditions exceeding 0.9, suggesting no necessary causal conditions for the occurrence of the two outcomes (see Table 4). No single precondition explains these results. Therefore, in this study, no causal conditions were excluded, and all preconditions were included in the subsequent analysis.

To explore the mechanisms influencing employees’ intrapreneurial behavior, this study constructs and analyzes the configuration of these intrapreneurial behaviors. Through the aforementioned data processing, a specific analysis was conducted on the configuration of intrapreneurial behavior among a group of high-performing and non-high-performing employees.

## 5. Results and Discussion

### 5.1. Configurational Analysis of High-Performing Employees’ Intrapreneurial Behavior

In the configurational analysis of high-performing employees’ intrapreneurial behavior, employees’ intrapreneurial behavior (FEB) was chosen as the outcome variable. The selection criteria were set to ensure consistency greater than 0.8 and a minimum case threshold of 4. The generated truth table was analyzed using fsQCA3.0 in order to obtain the configuration results of the factors influencing employees’ intrapreneurial behavior. Based on the intermediate solution of the final results, six configurations that can achieve employees’ intrapreneurial behavior were identified. The specific configuration paths are listed in Table 5.

According to the output results, the configuration of high intrapreneurial behavior among employees yielded six result paths with an overall coverage of 0.787. Thus, a combination of the preceding conditions in these six result paths can explain approximately 79% of the sample cases of intrapreneurial behavior in employees. Among these paths, the second has the highest coverage rate (0.673), indicating greater explanatory power for employees’ intrapreneurial behavior. Simultaneously, all combination paths have consistency values higher than the standard threshold of 0.8, with four exceeding 0.9. The overall consistency is 0.887, indicating a high level of consistency for the six preceding condition paths in explaining employees’ intrapreneurial behavior, making them significant contributing factors to this behavior.

In the two driving modes of high intrapreneurial behavior among employees, the first is the ability-driven mode, which includes Solution 1a (Entrepreneurial Competence * Task Valence * Perceived Value * Reward Mechanism), Solution 2a (Entrepreneurial Competence * Entrepreneurial Self-Efficacy * Management Support * Reward Mechanism), Solution 4a (Entrepreneurial Competence * Entrepreneurial Self-Efficacy * ~Task Valence * ~Perceived Value * Reward Mechanism), and Solution 6a (Entrepreneurial Competence * Entrepreneurial Self-Efficacy * Task Valence * ~Perceived Value * ~Management Support * ~Reward Mechanism). In all four paths, entrepreneurial competence serves as the core condition, playing a dominant role in explaining the paths, while the other variables serve as marginal conditions. In the configuration of Solution 1a, FEC * FTS * FPV * FRW indicates that when employees possess entrepreneurial competence, regardless of their level of entrepreneurial self-efficacy, as long as they have high task valence and perceived value and the company has corresponding reward mechanisms, employees can engage in intrapreneurial behavior. In the configuration of Solution 2a, FEC * FES * FMS * FRW indicates that when employees have both entrepreneurial competence and self-efficacy, and the company provides management support and reward mechanisms, employees’ intrapreneurial behavior can occur regardless of their levels of task valence and perceived value. In the configuration of Solution 4a, FEC * FES * ~FTS * ~FPV * FRW indicates that even with low levels of task valence and perceived value, and regardless of the presence of management support, employees with entrepreneurial competence and self-efficacy, along with some reward mechanisms in the company, can still exhibit intrapreneurial behavior. In the configuration of Solution 6a, FEC * FES * FTS * ~FPV * ~FMS * ~FRW indicates that when employees have entrepreneurial competence and self-efficacy and possess high levels of task valence, even with low perceived value and a lack of support and rewards from the company, they will engage in corresponding intrapreneurial behavior.

The second mode is the value-driven mode, which includes Solution 3a (Entrepreneurial Competence * Perceived Value * Management Support * Reward Mechanism) and Solution 5a (Entrepreneurial Self-Efficacy * Task Valence * Perceived Value * Management Support * Reward Mechanism). In both paths, perceived value and management support serve as core conditions, playing a dominant role in explaining the paths, whereas the other variables serve as marginal conditions.

In the configuration of Solution 3a, FEC * FPV * FMS * FRW indicates that when employees perceive high value in returns and the company fully supports employees’ intrapreneurial behavior, regardless of their level of entrepreneurial self-efficacy, as long as employees possess entrepreneurial competence and the company has corresponding reward mechanisms, they will spontaneously engage in intrapreneurial behavior. In the configuration of Solution 5a, ~FES * FTS * FPV * FMS * FRW indicates that even in the absence of entrepreneurial self-efficacy, if employees have high levels of task valence and perceived value, and the company provides support and rewards, employees’ intrapreneurial behavior can occur regardless of their level of entrepreneurial competence.

Based on the analysis of previous literature, high perceived value represents the overall value that employees attach to the rewards they receive for completing job tasks. Strengthening perceived value increases employees’ willingness to engage in intrapreneurial behavior. With the provision of management support by a company, obstacles to employees’ internal entrepreneurship are greatly reduced. The reward mechanism ensures that employees receive adequate rewards for completing tasks, thereby motivating those with entrepreneurial competence to actively engage in internal entrepreneurial activities and achieve value acquisition.

Simultaneously, employees who lack entrepreneurial self-efficacy have low confidence in engaging in innovative and entrepreneurial activities. Self-efficacy alone is not sufficient to promote employees’ intrapreneurial behavior. However, when task valence and perceived value are high, employees have strong expectations and perceptions of rewards. Additionally, the company provides extensive management support and sets corresponding reward mechanisms for employees’ intrapreneurial behavior, which largely satisfies their expectations for rewards after completing their work. Employees’ pursuit of value counterbalances the negative effects of their lack of self-efficacy and motivates them to attempt intrapreneurial behavior. It should be noted that in these two paths, employees’ pursuit of value and the company’s management support play core roles.

Among the six configurations, entrepreneurial competence appeared most frequently in five resulting paths. This is a core condition in configurations such as solutions 1a, 2a, 4a, and 6a. Perceived value and organizational management support appear to be the core conditions in the two result paths. This indicates that entrepreneurial competence, perceived value, and management support play crucial roles in determining whether employees engage in intrapreneurial behaviors.

According to the qualitative comparative analysis method, intrapreneurial behavior is not influenced by a specific combination of conditions but rather exists in multiple combined paths. In each result path, multiple factors work together to trigger the behavior, indicating the presence of multiple concurrent causal relationships among the factors. For example, in Solution 2a, intrapreneurial behavior is the result of the combined effects of entrepreneurial competence, self-efficacy, management support, and reward mechanisms.

The two types of high intrapreneurial behavior modes–ability-driven and value-driven–are formed by the combined effects of different combinations of antecedent conditions. The presence or absence of certain antecedent conditions can lead to intrapreneurial behavior. For example, entrepreneurial competence is a core condition in various result paths of the first behavior mode, but it may or may not be present in Solution 5a. This suggests that, although entrepreneurial competence has a positive facilitating effect on employees’ intrapreneurial behavior, the absence of entrepreneurial competence does not necessarily prevent employees from engaging in intrapreneurial behavior. This demonstrates the asymmetry of causal relationships.

### 5.2. Configurational Analysis of Non-High-Performing Employees’ Intrapreneurial Behavior

Based on the previous analysis, causal relationships in the qualitative comparative analysis exhibit asymmetry. The occurrence of results is not solely determined by a single linear relationship but requires different combinations of antecedent conditions to provide joint explanations. Therefore, the configurations derived in this study for high intrapreneurial behavior cannot be directly applied to non-high intrapreneurial behavior. Non-high intrapreneurial behavior is caused by specific combinations of antecedent conditions. Consequently, in the configuration analysis of non-high intrapreneurial behavior, the variable “non-high intrapreneurial behavior (~FEB)” is selected as the outcome variable, using the same consistency and case coverage thresholds as in the previous analysis. Results of the specific configuration path analysis are presented in Table 6.

According to the output results, configurations of antecedent conditions influencing non-high-performing employees’ intrapreneurial behavior construct five result paths, with an overall coverage of 0.669. Thus, combinations of antecedent conditions in these five resulting paths can explain approximately 67% of the cases of non-high-performing employees’ intrapreneurial behavior. The first path has the highest coverage rate of 0.514, indicating greater explanatory power for non-high-performing employees’ intrapreneurial behavior. The consistency of these five paths is higher than the standard value of 0.8, with an overall consistency of 0.895. This finding suggests that these five configurations of antecedent conditions exhibit a high level of consistency in contributing to the occurrence of non-high-performing employees’ intrapreneurial behavior, making them important factors in explaining such behavior. This study categorizes these five result paths into three patterns that influence non-high-performing employees’ intrapreneurial behavior (Table 6).

The influence configuration of employees’ non-high intrapreneurial behavior consists of five result paths and three patterns. To gain a deeper understanding of the influence mechanism of employees’ intrapreneurial behavior, this study analyzes and discusses the influence configuration of employees’ non-high intrapreneurial behavior.

The first pattern is the ability hindrance type, which includes two paths: Solution 1b (entrepreneurial competence * entrepreneurial self-efficacy * perceived value * management support) and Solution 2b (entrepreneurial competence * entrepreneurial self-efficacy * task significance * reward mechanism). In the configuration of Solution 1b, FEC * FES * FPV * FMS indicates that when employees lack entrepreneurial competence, self-efficacy, and perceived value, and when the organization lacks management support, they will inhibit intrapreneurial behavior regardless of the level of task significance or the presence of a reward mechanism. In Solution 2b, the configuration FEC * FES * FTS * FRW indicates that even if employees have a high level of task significance and the organization implements a reward mechanism to motivate them, they will not engage in intrapreneurial behavior if they lack entrepreneurial competence and self-efficacy.

In both results, entrepreneurial competence plays a central role in explaining behavior, leading to naming this pattern as the ability-hindrance type. Based on the previous literature, entrepreneurial competence and self-efficacy are important factors that influence an individual’s ability and cognitive levels. When these two factors are lacking, employees tend to have pessimistic evaluations of their ability to engage in intrapreneurial behavior, believing that they are unlikely to successfully complete such tasks. Their expectations for task completion are very low, and, with the lack of a high level of perceived value, they start devaluing their work in terms of its importance. As a result, the subjective value of internal entrepreneurial work for employees decreases, and, coupled with the absence of management support, employees strongly inhibit intrapreneurial behavior. Even in the presence of high task significance and a reward mechanism, if the core factor of entrepreneurial competence is lacking, employees experience lower levels of motivation, leading to a significant reduction in their engagement in intrapreneurial behavior.

The second pattern is the perception hindrance type, which includes Solution 3b (entrepreneurial self-efficacy * task significance * perceived value * management support * reward mechanism). In the configuration of Solution 3b, FES * FTS * FPV * FMS * FRW indicates that even if employees possess entrepreneurial competence, in the absence of entrepreneurial self-efficacy and management support for intrapreneurial behavior, they will not engage in intrapreneurial behavior, despite high task significance and perceived value. In this path, entrepreneurial self-efficacy and management support are the core variables with primary explanatory roles.

Based on the relevant literature analysis, entrepreneurial self-efficacy is the self-assessment of an individual’s belief in their ability to engage in intrapreneurial behavior. Low levels of self-efficacy reduce employees’ willingness to engage in intrapreneurial behavior. In addition, unlike individual entrepreneurship, intrapreneurial behavior within an organization relies heavily on organizational support. When employees lack the intention to engage in internal entrepreneurship and are unable to obtain management support from the organization, they become more cautious in their evaluation of the task. Hence, their expectations from the task will decrease. Under the dual negative influence of individual and organizational factors, it becomes difficult to generate strong driving forces, and employees develop a sustained perception of low motivation for intrapreneurial behavior.

The third pattern is the value-hindrance type, which includes solutions 4b (entrepreneurial self-efficacy * task significance * perceived value * management support * reward mechanism) and 5b (entrepreneurial competence * entrepreneurial self-efficacy * task significance * perceived value * management support * reward mechanism). In the configuration of Solution 4b, FES * FTS * FPV * FMS * FRW indicates that regardless of the level of entrepreneurial competence, in the presence of low perceived value and lack of management support, employees will not engage in intrapreneurial behavior despite possessing a certain level of entrepreneurial self-efficacy and task significance, and in the absence of a reward mechanism. In the configuration of Solution 5b, FEC * FES * FTS * FPV * FMS * FRW indicates that under the same conditions of low perceived value and lack of management support, if employees have high levels of competence and perception but lack task significance, they will not engage in internal entrepreneurship even in the presence of corresponding incentives. In these two result paths, perceived value and management support are the core conditions that play primary explanatory roles.

Reviewing the relevant literature shows that a lack of perceived value for internal entrepreneurship results in employees having a low level of perceived value for their work. Internal entrepreneurial activities lack sufficient attractiveness to employees, and the inability of the organization to provide support for employees’ intrapreneurial behavior reduces their interest in internal entrepreneurship. Even if employees believe that they are capable of internal entrepreneurship, their perceptions of the value of the task remain low. Under the influence of these core factors, employees’ perceptions of the task’s significance will be low, and the driving force generated will be reduced. Consequently, employees are inhibited from engaging in intrapreneurial behavior.

## 6. Conclusions

Based on expectancy and person-environment fit theories, this study identifies and summarizes six antecedent conditions that influence employees’ intrapreneurial behavior: entrepreneurial competence, self-efficacy, task significance, perceived value, management support, and reward mechanisms. Using the theoretical “expectancy-value-motivation” framework, this study constructs an analytical framework to examine the mechanisms influencing employees’ internal entrepreneurship. Existing validated scales were used to collect, screen, and organize the data. The data were then recalibrated using qualitative comparative analysis. After conducting a necessary analysis of the antecedent conditions, a truth table was constructed to analyze the configuration of high and non-high intrapreneurial behaviors. This study discusses multiple concurrent factors and causal mechanisms that influence employees’ intrapreneurial behavior.

The analysis revealed six paths for high intrapreneurial behavior. Based on the core factors, these paths were categorized into two influencing patterns: ability- and value-driven. Similarly, five result paths for non-high intrapreneurial behavior were categorized into three influencing patterns: ability hindrance, perception hindrance, and value hindrance, based on different core factors. By discussing and analyzing the configurations of the six antecedent conditions for high intrapreneurial behavior and five configurations for non-high intrapreneurial behavior, this study arrived at its main conclusions.

Special attention should be paid to the influence of entrepreneurial competence and perceived value factors on employees’ intrapreneurial behavior. An analysis of the six result paths of high intrapreneurial behavior revealed that entrepreneurial competence, perceived value, and management support play core influential roles in each path, while entrepreneurial self-efficacy, task significance, and reward mechanism appear as peripheral conditions in various result paths. Among the three core conditions, entrepreneurial competence and perceived value are important individual-level influencing factors, whereas management support is an organizational-level influencing factor. This indicates the crucial role of individual factors in driving internal entrepreneurial processes. Studies underscore the pivotal function of personal initiative in fostering intrapreneurial actions and converting employees’ conduct into intrapreneurial initiatives [48].Employees with entrepreneurial competence demonstrate exceptional abilities in terms of opportunity identification, operational management, and other skills. Employees with this trait are more likely to discover market opportunities, and better understand subsequent tasks in the internal entrepreneurial process. The high perceived value of work ensures employees’ enthusiasm and willingness to engage in the work, and they are more willing to invest time and energy in intrapreneurial behavior. Under self-motivation, employees actively seek organizational support to engage in internal entrepreneurial activities. Thus, entrepreneurial competence and perceived value are important intrinsic factors that influence employees’ intrapreneurial behavior.

The impact of management support on employees’ intrapreneurial behavior should be studied. Results of high intrapreneurial behavior show that management support, as a core factor at the organizational level, significantly influences employees’ engagement in internal entrepreneurship. According to person-environment fit theory, individual behavior is influenced not only by individual factors but also by the interaction between individual and environmental factors. In the context of internal entrepreneurship, employees exist within an organization and operate in the company’s management environment. All these actions are influenced by the organizational environment. Internal entrepreneurship, which involves working outside employees’ primary responsibilities, poses challenges when employees lack support from management. It becomes difficult for employees to acquire resources, and they face increasing concerns and pressure while balancing their primary work and internal entrepreneurial activities. When a company fully supports employees in its internal entrepreneurial endeavors, it fosters a positive atmosphere that encourages and supports employee innovation and entrepreneurship. This motivates employees to engage in internal entrepreneurship wholeheartedly and alleviates their concerns about potential drawbacks. At the organizational level, a conducive structure can encourage employees’ involvement in intrapreneurial activities. Studies indicate that an organizational framework that includes managerial backing and job autonomy can stimulate employees’ engagement in intrapreneurial pursuits [49,50]. According to the employee expectancy theory, employees’ motivation for work is determined by their expectations and valence. Management support has a positive impact on outcomes, and when it is substantial, it can, to some extent, compensate for deficiencies in other factors, such as a lack of entrepreneurial self-efficacy. The absence of management support does not necessarily imply that employees do not engage in internal entrepreneurship. However, in the absence of this core condition, if employees still desire to engage in internal entrepreneurship, the requirements for the other prerequisite factors become more stringent. Therefore, management support, as a core factor influencing employees’ intrapreneurial behavior, plays a significant role in promoting enthusiasm for internal entrepreneurship.

Focus should be placed on the configuration effects of multiple factors. A qualitative comparative analysis based on fuzzy sets reveals that employees’ intrapreneurial behavior is influenced by multiple antecedent variables working together, with no single unique path leading to the final outcome. This suggests that different combinations of antecedent factors can result in the same outcomes to some extent. It indicates that the absence or low levels of certain antecedent conditions do not necessarily imply a lack of intrapreneurial behavior. Furthermore, an analysis of the result paths of high intrapreneurial behavior and low intrapreneurial behavior shows clear asymmetry between the two. The factors leading to intrapreneurial behavior are not simply the opposite of those leading to low intrapreneurial behavior. Therefore, in the research process, it is necessary to consider the configuration effects of antecedent conditions and their impact on the outcomes. Only by doing so can we conduct a more thorough and in-depth investigation of employees’ intrapreneurial behavior.

This study examines the mechanisms influencing employees’ intrapreneurial behavior by considering various factors at both the individual and organizational levels. It identifies multiple paths that either promote or inhibit employees’ intrapreneurial behavior. Studies propose a linkage between individual and organizational elements. Employee perceived entrepreneurial self-efficacy leads to entrepreneurial behavior within the firm [51]. The degree to which employees can tap into their creativity for opportunity identification hinges on various aspects like organizational design, how firms organize their intrapreneurial processes, and the interactions among individuals within the organization. Up to now, these research domains have been underexplored and therefore warrant additional scrutiny [52]. By reviewing the relevant literature, this study clarifies the conceptual meanings of internal entrepreneurship and intrapreneurial behavior within the current research field. It traces the developmental history of core concepts and affirms Neessen’s comprehensive concept of internal entrepreneurship proposed in 2019. Internal entrepreneurship refers to the process by which employees identify and exploit opportunities through innovation, proactivity, and risk-taking. This process enables organizations to create new products, processes, and services, leading to self-renewal or entrepreneurial activities that enhance competitiveness and performance [5]. A corporate culture that empowers employees to chase their personal goals and convictions alongside the organization’s values can enhance job contentment and efficiency, while also establishing a foundation for intrapreneurial endeavors [49]. Intrapreneurial activities tend to rely more heavily on organizational resources and influences compared to other types of work behaviors. These traits have prompted additional requests for research that explores the interconnectedness between intrapreneurial behaviors and organizational attributes [52,53]. The occurrence of employees’ intrapreneurial behavior is complex, and existing research on this topic utilizes traditional linear regression analysis to examine the causal relationships among employees’ intrapreneurial behavior. However, this approach overlooks the asymmetry and complexity of causal relationships, resulting in research models that fail to fully explain the diversity of employees’ intrapreneurial behaviors. This study employed a qualitative comparative analysis approach to calibrate the research variables as fuzzy-set membership degrees. This allows us to examine the causal relationships between various antecedent factors and employees’ intrapreneurial behavior. The resulting multiple paths of influence demonstrate equivalence, implying that different paths can lead to the same outcome. Additionally, a comparison between the configuration of high intrapreneurial behavior and non-high intrapreneurial behavior among employees confirms the asymmetric nature of the causal relationships in employees’ intrapreneurial behavior. This further enhances the explanatory power of research on the mechanisms that influence employees’ intrapreneurial behavior.

## 7. Limitations

Although this study provides a new research perspective on the intrapreneurial behavior of employees, there are still some shortcomings due to the limited use of this method in the field and the existence of various influencing factors. These need to be addressed and improved in future work. The specifics include the following aspects. First, in terms of questionnaire design, the selected antecedent variables cannot cover all the conditional factors that influence employee behavior. In addition to the six influencing factors listed in this study, there are other factors that affect intrapreneurial behavior of employees that this paper does not touch upon. Moreover, although the scales used in this study are mature, there may still be some incompleteness in the measurement dimensions of the variables. For example, in addition to opportunity recognition ability, operational management ability, and stress resistance ability, are there other dimensions to entrepreneurial competence? Future research can analyze the antecedent factors affecting the intrapreneurial behavior of employees from different levels and perspectives, in order to enrich the research content in the field of intrapreneurship. Second, in terms of data collection, this study distributed questionnaires and collected research data through a questionnaire data collection website.so future research can further improve the data collection method, pay attention to the quality of questionnaire completion, such as using on-site completion, on-site interviews and other more intuitive and manageable methods, in order to obtain more accurate data.

## Figures and Tables

**Table 1:** Reliability Test.

Variable	Number of Items	Partial Cronbach’s α	Overall Cronbach’s α
Entrepreneurial Competence	16	0.9	0.9
Entrepreneurial Self-Efficacy	9	0.8	
Task Valence	3	0.8	
Perceived Value	3	0.7	
Management Support	4	0.8	
Reward Mechanism	3	0.8	
Employees’ intrapreneurial Behavior	9	0.8	

**Table 2:** Convergent and Discriminant Validity of the Construct Measures.

Variable	FL	CR	AVE
Entrepreneurial Competence	0.6–0.8	0.9	0.5
Entrepreneurial Self-Efficacy	0.6–0.8	0.8	0.5
Task Valence	0.7–0.8	0.8	0.6
Perceived Value	0.7–0.8	0.7	0.6
Management Support	0.6–0.7	0.8	0.5
Reward Mechanism	0.6–0.7	0.7	0.5
Employees’ intrapreneurial Behavior	0.7–0.8	0.9	0.6

**Table 3:** KMO Value and Bartlett’s Sphericity Test Results for Factors Influencing Employees’ intrapreneurial Behavior.

KMO Value		0.893
Bartlett’s Sphericity Test	Approx. Chi-Square	4119.342
	Degrees of Freedom	741
	Significance	0.000

**Table 4:** Results of Necessary Condition Analysis for Single Variables.

Antecedent Variable	High-Performing Employees’ Intrapreneurial Behavior		Non-High-Performing Employees’ Intrapreneurial Behavior	
	Consistency	Coverage	Consistency	Coverage
FEC	0.892767	0.842275	0.460572	0.362386
~FEC	0.324163	0.418796	0.79954	0.861462
FES	0.824951	0.807867	0.479609	0.391703
~FES	0.378841	0.466073	0.764749	0.784644
ETS	0.641074	0.629528	0.621646	0.509105
~ETS	0.500102	0.613139	0.547632	0.559947
FPV	0.813181	0.770223	0.517764	0.408996
~FPV	0.376036	0.483206	0.709116	0.759938
FMS	0.863683	0.815257	0.476983	0.375492
~FMS	0.338398	0.436877	0.765323	0.824014
FRW	0.804352	0.764189	0.541395	0.42897
~FRW	0.39896	0.510553	0.702387	0.749628

**Table 5:** Configuration Analysis of High Intrapreneurial Behavior among Employees.

Antecedent Conditions	Solution					
	H1a	H2a	H3a	H4a	H5a	H6a
Entrepreneurial Competence	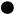	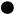	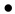	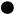		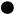
Entrepreneurial Self-efficacy		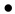		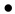	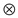	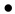
Task Efficacy	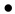			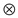	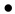	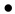
Perceived Value	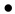		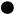	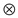	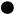	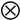
Managerial Support		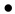	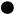		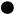	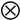
Reward Mechanism	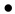	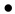	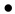	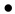	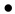	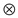
Consistency	0.926954	0.945296	0.924856	0.956735	0.840138	0.813071
Raw coverage	0.471668	0.67345	0.634887	0.22729	0.173134	0.103196
Overall solution coverage	0.786803					
Overall solution consistency	0.887232					

Note: In the table, “
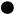
” represents the presence of a condition as a core condition, “
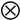
” represents the absence of a condition as a core condition, “
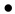
” represents the presence of a condition as a peripheral condition, “
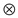
” represents the absence of a condition as a peripheral condition, and a blank space indicates that the presence or absence of a condition has no impact on the occurrence of the outcome. If all preceding conditions are blank, the configuration is considered a logical remainder.

**Table 6:** Configuration Analysis of non-High Intrapreneurial Behavior among Employees.

Antecedent Conditions	Solution				
	H1b	H2b	H3b	H4b	H5b
Entrepreneurial Competence	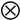	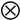			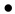
Entrepreneurial Self-efficacy	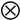	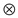	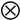	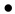	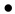
Task Efficacy		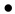	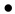	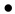	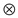
Perceived Value	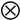		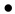	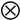	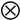
Managerial Support	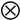		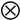	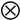	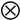
Reward Mechanism		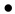	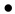	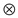	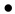
consistency	0.946849	0.878625	0.895389	0.912355	0.879866
Raw coverage	0.513779	0.234134	0.164565	0.145913	0.132687
Overall solution coverage	0.668511				
Overall solution consistency	0.894906				

Note: In the table, “
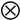
” represents the absence of a condition as a core condition, “
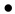
” represents the presence of a condition as a peripheral condition, “
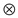
” represents the absence of a condition as a peripheral condition, and a blank space indicates that the presence or absence of a condition has no impact on the occurrence of the outcome. If all preceding conditions are blank, the configuration is considered a logical remainder.

## Data Availability

The datasets generated and analyzed during the study are available from the corresponding author upon reasonable request.
